# NH_3_-treated WO_3_ as low-cost and efficient counter electrode for dye-sensitized solar cells

**DOI:** 10.1186/s11671-014-0708-z

**Published:** 2015-01-28

**Authors:** Dandan Song, Zhao Chen, Peng Cui, Meicheng Li, Xing Zhao, Yaoyao Li, Lihua Chu

**Affiliations:** State Key Laboratory of Alternate Electrical Power System with Renewable Energy Sources, School of Renewable Energy, North China Electric Power University, No. 2 Beinong Rd, Changping, Beijing, 102206 China; Suzhou Institute, North China Electric Power University, No. 377 Linquan Rd, Dushuhu, Suzhou, 215123 China

**Keywords:** Tungsten trioxide (WO_3_), NH_3_ treatment, Counter electrode, Catalytic, Power conversion efficiency

## Abstract

A novel low-cost and efficient counter electrode (CE) was obtained by treating catalytic inert tungsten trioxide (WO_3_) nanomaterial in NH_3_ atmosphere at elevated temperatures. The formation of tungsten oxynitride from WO_3_ after NH_3_ treatment, as evidenced by X-ray photoelectron spectroscopy and X-ray diffraction, increases the catalytic activity of the CE. Correspondingly, the power conversion efficiency (PCE) of the DSC is significantly increased from 0.9% for pristine WO_3_ CE to 5.9% for NH_3_-treated WO_3_ CE. The photovoltaic performance of DSC using NH_3_-treated WO_3_ CE is comparable to that of DSC using standard Pt CE (with a PCE of 6.0%). In addition, it is also shown that NH_3_ treatment is more efficient than H_2_ or N_2_ treatment in enhancing the catalytic performance of WO_3_ CE. This work highlights the potential of NH_3_-treated WO_3_ for the application in DSCs and provides a facile method to get highly efficient and low-cost CEs from catalytic inert metal oxides.

## Background

Dye-sensitized solar cells (DSCs) have attracted great attention for their low cost, simple production, and acceptable energy conversion efficiency [1,2]. It typically consists of three parts: a dye-sensitized oxide layer, electrolyte, and a counter electrode (CE). As an important component of DSCs, the CE transfers the electrons from the external circuit to the internal electrolyte and thus reduces triiodide ions to iodide ions, which realizes the continuous operation of DSCs and greatly influences the photovoltaic performance of DSCs. For achieving the high performance of DSCs, the CEs should possess high conductivity and catalytic activity [3]. High catalytic active platinized fluorine-doped tin oxide (FTO) is the most commonly used CE in DSCs. However, the high cost of scarce Pt limits the large-scale fabrication and application of DSCs, which promotes the exploration of Pt-free CEs [3-5].

Carbonaceous materials [6], conducting polymers [7], inorganic compounds (like sulfides [8], carbides [9], and nitrides [10]), and composite materials [11-13] have been reported as Pt-free materials in DSCs. The metal oxides were also studied as CEs for their facile synthesis and low cost, but the efficiencies were relatively low and not able to replace Pt [3]. These oxides may be further improved by changing their electronic structure. Hydrogen (H_2_) or nitrogen (N_2_) treatments have been proved to be a facile and efficient method to change the electronic structure of oxides, with which the efficiencies were improved from 0.63% to 5.43% for WO_3_ by H_2_ treatment and from 1.84% to 6.09% for SnO_2_ by N_2_ treatment [14,15]. However, the DSCs using these CEs still yield low fill factors (FF) and low efficiencies as compared to conventional Pt CEs; further improvements need to be carried out.

In this work, we demonstrated that the electronic structure of the metal oxide (WO_3_) was able to be facilely changed by NH_3_ treatment and its catalytic activity was also improved. The DSC using NH_3_-treated WO_3_ exhibits superior photovoltaic performance with a power conversion efficiency (PCE) of 5.9%, which is similar to that using standard Pt CE (6.0%) and is much higher than that using pristine WO_3_ CE (0.9%). Moreover, we also demonstrated that NH_3_ treatment was more efficient than H_2_ or N_2_ treatment in improving the performance of DSCs using WO_3_-based CEs.

## Methods

### Preparation of WO_3_, NH_3_-treated WO_3_, and standard Pt CEs

The original WO_3_ nanopowders are commercial products with a particle diameter of about 30 nm. To prepare the WO_3_ slurry, 133 mg WO_3_ and 20 mg ethyl cellulose are dispersed in 1 ml alpha-terpineol and then stirred for 24 h to form a fluid mixture. The yellow-green slurry was deposited on pre-cleaned FTO/glass substrates by doctor blade method to form continuous films. The films were then dried at 110°C for 30 min to remove the organic solvents and the WO_3_ CEs were obtained. Atmosphere (including NH_3_, H_2_, and N_2_)-treated WO_3_ CEs were obtained by annealing the as-prepared WO_3_ CEs in different atmospheres at 480°C for 2 h. Standard Pt CE was also fabricated by sputtering thermodecomposition of H_2_PtCl_6_ on pre-cleaned FTO/glass at 450°C for 20 min.

### Fabrication of DSCs

TiO_2_ films were prepared by doctor blading of TiO_2_ nanoparticle (P25) slurry on FTO/glass substrates. All of the TiO_2_ films were post-treated with TiCl_4_. After calcination, the TiO_2_ films were immersed in a 0.3 mmol/l ethanol solution of N719 dye for 24 h. The DSCs were fabricated by assembling dye-sensitized TiO_2_ photoanodes with as-fabricated CEs using 30-μm-thick Surlyn (DuPont, Wilmington, DE, USA). I^−^/I_3_^−^ electrolyte with acetonitrile as the solvent was used. The active area of solar cells was about 4 mm × 4 mm. Symmetric cells for electrochemical measurements were fabricated by assembling two identical CEs together using 30-μm-thick Surlyn.

### Characterization methods

The structure and morphology properties of the samples were measured by X-ray diffraction (XRD; XRD-6000, Shimadzu Corp., Kyoto, Japan) and scanning electron microscopy (SEM; S-4800, Ltd., Tokyo, Japan). The element distribution was tested by X-ray photoelectron spectroscopy (XPS) and electron diffraction spectroscopy (EDS). The photovoltaic performance of DSCs was characterized using a source meter (2400, Keithley Instruments, Inc., Beijing, China) under AM 1.5G irradiation (100 mW/cm^2^) generated by a solar simulator (XES-301S + EL-100, San-ei Electric Co., Ltd., Osaka, Japan). Electrochemical impedance spectroscopy (EIS) was carried out using the electrochemical workstation (CHI660D), performed on symmetric cells.

## Results and discussion

**Figure 1:**
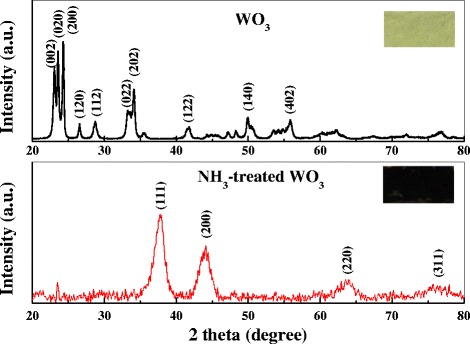
**XRD curves of pristine WO**_**3**_ **and NH**_**3**_ **-treated WO**_**3**_ **samples.** The insets show the digital pictures of WO_3_ and NH_3_-treated WO_3_ powders.

Figure 1 shows the XRD patterns of the pristine WO_3_ and NH_3_-treated WO_3_ products. The most intensive diffraction peaks of the pristine WO_3_ match well with the typical monoclinic WO_3_ (JCPDS no. 431035). However, after NH_3_ treatment, the locations of the intensive diffraction peaks are totally changed, which match well with the tungsten oxynitride (WO*x*N*y*, JCPDS no. 251254). Correspondingly, the color of WO_3_ is also changed from yellow to black after NH_3_ treatment (insets of Figure 1).

**Figure 2:**
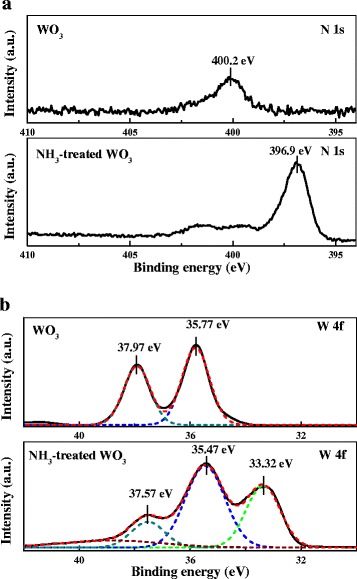
**N 1 s (a) and W 4f (b) XPS spectra of WO**_**3**_ **and NH**_**3**_ **-treated WO**_**3**_ **samples.** The solid lines represent the measured data, and the dashed lines represent the Gaussian fitted curves.

However, as cubic tungsten nitride (WN, JCPDS no. 751012) and WO*x*N*y* (JCPDS No. 251254) have almost identical lattice structures and hence diffraction peaks in the XRD pattern, it is difficult to distinguish them only with XRD results [16-18]. Hence, the surface chemical element composition was studied by XPS. Figure 2a shows the N 1 s XPS spectra of WO_3_ and NH_3_-treated WO_3_ samples. In the condition of WO_3_, the low-intensity and relatively broad peak at 400.2 eV can be ascribed to the γ-N state caused by chemisorbed nitrogen molecules on the WO_3_ surface [19]. In the condition of NH_3_-treated WO_3_, the high-intensity peak at 396.9 eV can be observed, which corresponds to the β-N state and is essentially the atomic N [18,19], demonstrating that nitrogen has been successfully incorporated into the WO_3_.

The W 4f XPS spectra of WO_3_ and NH_3_-treated WO_3_ samples are shown in Figure 2b. The peaks at 35.77 eV (W 4f_7/2_) and 37.97 eV (W 4f_5/2_) from WO_3_ can be ascribed to the binding energy of high oxidation state of W. In comparison, one additional peak at 33.32 eV (W4f_7/2_), which is associated with lower oxidation states of W, can be observed from the NH_3_-treated WO_3_ sample, indicating the formation of W-N bonds in NH_3_-treated WO_3_ as might be expected in tungsten oxynitrides [18]. In addition, the peaks located at 35.37 and 37.47 eV from NH_3_-treated WO_3_ are lower compared with those from the pristine WO_3_ (35.77 and 37.97 eV), which probably result from the existence of less electronegative atoms into the oxide lattice considering the fact that N has smaller electronegativity (3.04) than O (3.44). From the above results, it can be concluded that WO*x*N*y*, other than tungsten nitrides, were formed, as in good accordance with the previous XRD analysis.

**Figure 3:**
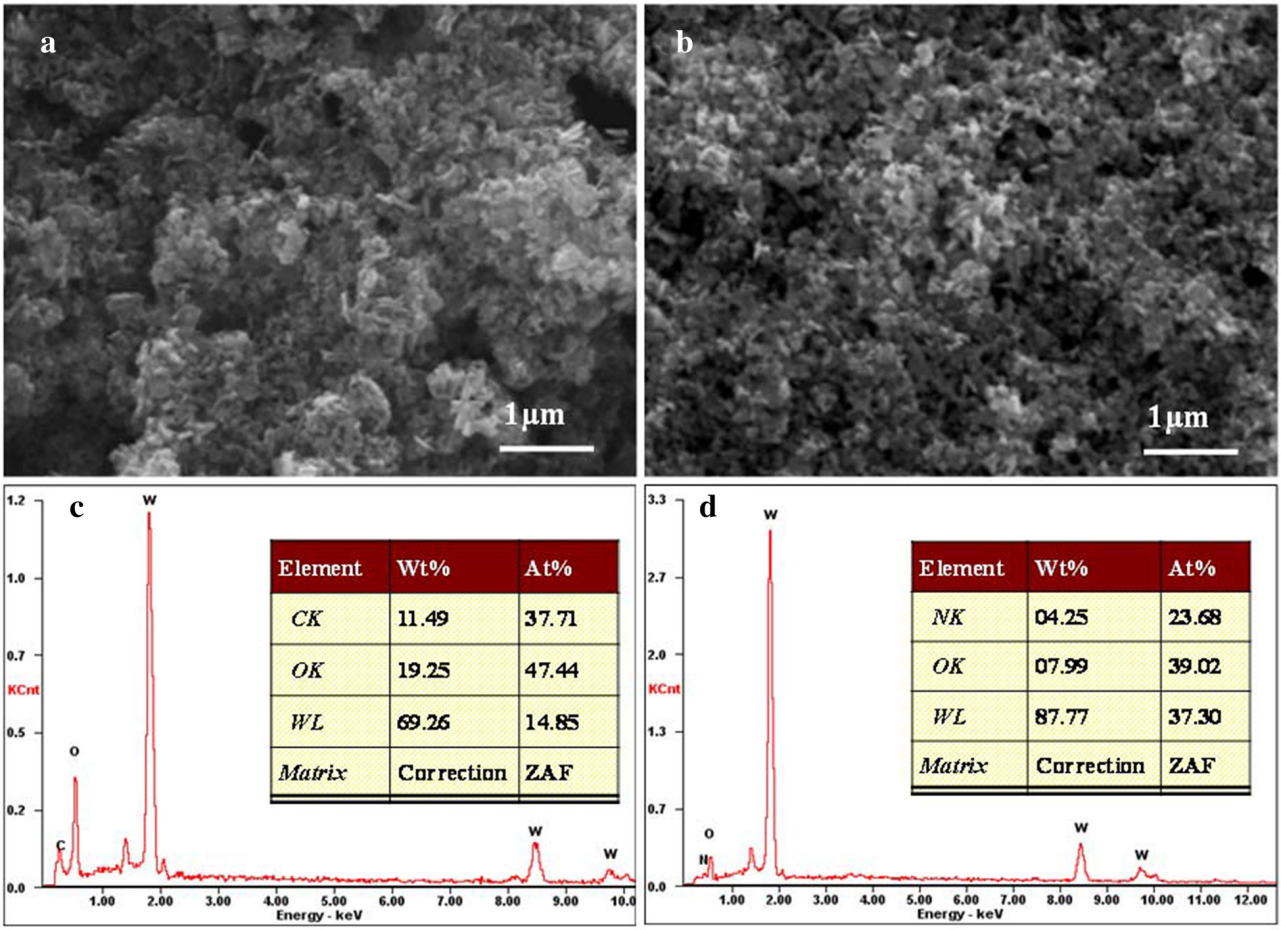
**SEM images (a, b) and EDS spectra (c, d) of WO**_**3**_ **CE and NH**_**3**_ **-treated WO**_**3**_ **CE.** Insets in **(c)** and **(d)** are the corresponding detailed elemental distribution.

The morphology of the two different WO_3_ CEs was also characterized by SEM. Figure 3a,b presents the top-view SEM images of WO_3_ and NH_3_-treated WO_3_ CE, respectively. It is clear that these two CEs are both porous which is useful for the diffusion of iodide/triiodide redox couples in the films. The EDS patterns shown in Figure 3c,d from these two CEs are quite different. No signal of N can be observed in WO_3_ CE (Figure 3c), while the signal of N is obvious in NH_3_-treated WO_3_ (Figure 3d). In addition, the atomic ratio of O to W is decreased from 3.19 to 1.05 by NH_3_ treatment, suggesting that the oxygen sites are partially substituted by nitrogen atoms in reductive NH_3_ atmosphere.

**Figure 4:**
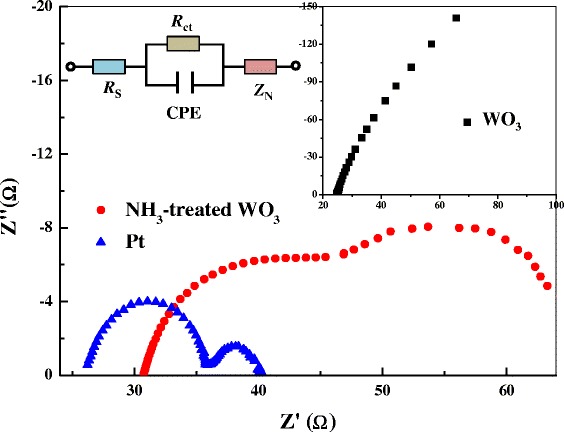
**Nyquist plots from symmetric cells with different counter electrodes.** The inset shows the equivalent circuit of the symmetric cells.

To study the kinetics of the catalytic property of the CEs, EIS was carried out on symmetric cells fabricated with two identical CEs. Nyquist plots from WO_3_, NH_3_-treated WO_3_, and standard Pt CEs are shown in Figure 4, and the equivalent circuit of the symmetric cells is shown in the inset of Figure 4. The high-frequency intercept at the real axis (*Z*′) represents the series resistance (*R*_S_). Two arcs can be seen in the Nyquist plots, which correspond to the charge transfer resistance (*R*_CT_) and the capacitance (CPE) at electrolyte/electrode interface (the left arc in the high-frequency region) and the Nernst diffusion impedance (*Z*_N_) of redox sites in the electrolyte (the right arc in the low-frequency region), respectively [3,10,12]. The simulated *R*_CT_ of the NH_3_-treated WO_3_ CEs is 9.2 Ω, similar to that of the Pt electrode (9.3 Ω). In regard to the pristine WO_3_ CE, the electrocatalytic activity is lower according to its large *R*_CT_ (>100 Ω). The simulated *Z*_N_ of Pt CE is 4.7 Ω, while those of WO_3_ CE and NH_3_-treated WO_3_ CE are higher probably due to combination of the Nernst diffusion impedance and the porous diffusion impedance in the porous WO_3_-based CEs. Nevertheless, the similar *R*_CT_ value of NH_3_-treated WO_3_ and standard Pt CE highlights the superior electrocatalytic activity of NH_3_-treated WO_3_ CE for the reduction of triiodide ions, which provides a crucial precondition for replacing the Pt CE with the NH_3_-treated WO_3_ CE in DSCs.

**Figure 5:**
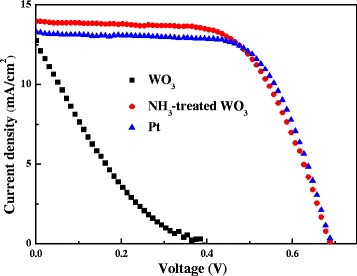
**Photocurrent density-voltage curves of DSCs using WO**_**3**_ **, NH**_**3**_ **-treated WO**_**3**_ **, and standard Pt CEs under 100 mW/cm** ^**2**^ **irradiation.**

Figure 5 presents the photocurrent density-voltage (*J*-*V*) curves of the DSCs using WO_3_, NH_3_-treated WO_3_, and standard Pt CEs. The detailed photovoltaic parameters from the *J*-*V* curves are summarized in Table 1. The DSC with WO_3_ CE has a poor photovoltaic performance with a low FF of 17.6% and a low short-circuit current density (*J*_sc_) of 12.8 mA/cm^2^. With NH_3_ treatment, the related DSC shows an improved photovoltaic performance with a FF of 62.0% and a *J*_sc_ of 14.0 mA/cm^2^. Therefore, the PCE of DSC using NH_3_-treated WO_3_ CE (5.9%) is much higher than that of DSC using pristine WO_3_ CE (0.9%). In comparison, the DSC with standard Pt CE has also been characterized and shows a similar PCE (6.0%) to that with NH_3_-treated WO_3_ CE, demonstrating the potential of NH_3_-treated WO_3_ CEs used as Pt substituents.

**Table 1:** **Photovoltaic parameters of DSCs using WO**_**3**_ **, NH**_**3**_ **-treated WO**_**3**_ **, and standard Pt CE**

**CEs**	***J***_**sc**_ **(mA/cm** ^**2**^ **)**	***V***_**oc**_ **(V)**	**FF (%)**	**PCE (%)**
WO3	12.8	0.40	17.6	0.9
NH3-treated WO3	14.0	0.68	62.0	5.9
Pt	13.3	0.69	65.4	6.0

**Figure 6:**
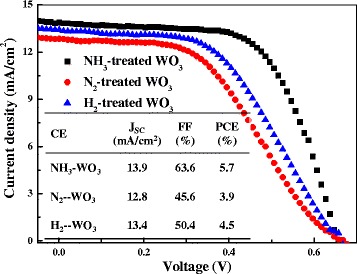
**Photocurrent density-voltage curves of DSCs using WO**_**3**_ **CEs treated under different atmospheres under 100 mW/cm** ^**2**^ **irradiation.**

It is worth noting that the FF and PCE obtained from DSC using the NH_3_-treated WO_3_ CE are also relatively higher in comparison with those from DSC using tungsten nitrides [10]. In addition, the obtained FF and PCE from DSC using NH_3_-treated WO_3_ CE are also higher than those from DSC using H_2_- or N_2_-treated WO_3_ CE. The N_2_- and H_2_-treated WO_3_ were also fabricated under the same conditions with NH_3_-treated WO_3_ and used as CEs for DSCs. As shown in Figure 6, the DSC using N_2_-treated WO_3_ CE yields a lower FF of 45.6% and a PCE of 3.9%. The DSC using H_2_-treated WO_3_ CE shows a FF of 50.4% which is similar to previous report [14] and a PCE of 4.5%. The DSC using NH_3_-treated WO_3_ CE exhibits the best performance with the highest FF and PCE, demonstrating the great advantage of NH_3_ treatment for preparing highly efficient and low-cost CEs.

The excellent performance of NH_3_-treated WO_3_ CE can be ascribed to the change of electronic structure from tungsten oxide to tungsten oxynitride by NH_3_ treatment. NH_3_-treated WO_3_ CE possesses similar W-N bonds to tungsten nitride which is a catalytic active site for the reduction of triiodide [10,20]; hence, it is also able to provide Pt-like electrocatalytic properties. Meanwhile, as the reduction ability of NH_3_ also provides a reduction atmosphere for WO_3_, which will create oxygen vacancies as similar to the case of H_2_ treatment [14], the catalytic activity can also be improved in the presence of oxygen vacancies. Therefore, NH_3_-treated WO_3_ CE exhibits the best performance among the WO_3_ CEs treated in different atmospheres.

Moreover, NH_3_ treatment may also vary the energy level of WO_3_ by introducing oxygen vacancies. As the conduction band level of WO_3_ (approximately 0.7 V versus normal hydrogen electrode (NHE)) is larger than the potential of I^−^/I_3_^−^ (approximately 0.3 V versus NHE), the overpotential for triiodide reduction in WO_3_ CE will be inevitable, leading to a low *V*_OC_ in DSCs using WO_3_ CE (as shown in Figure 5). However, the *V*_OC_ values of DSCs using Pt CE and NH_3_-treated WO_3_ CE are nearly identical, which indicates that the overpotential for triiodide reduction in NH_3_-treated WO_3_ CE is negligible and the Fermi level of WO_3_ is varied by NH_3_ treatment. It is proposed that hydrogen incorporation in WO_3_ favors the occupation of gap states near the Fermi level and the maintenance of a high work function, which facilitate the charge transport and enhance charge extraction in organic solar cells [21]. NH_3_ treatment may also play a similar role in affecting the electronic structure of WO_3_ and can be explored as a hole-extracting layer for organic solar cells.

## Conclusions

In conclusion, it is demonstrated that NH_3_ treatment can significantly improve the catalytic performance of WO_3_ in the use of CE material for DSCs. By annealing commercial WO_3_ in a NH_3_ atmosphere, the oxygen atoms in WO_3_ can be partially substituted by nitrogen to form tungsten oxynitrides, which obviously enhance the catalytic activity of the CEs. Correspondingly, the DSC using NH_3_-treated WO_3_ CE exhibits excellent performance, which is comparable to the DSC using standard Pt CE. The findings in this work also provide new insights into the exploration of low-cost and highly efficient CE materials with metal oxynitrides for DSCs.
